# Suspected idiopathic sclerosing orbital inflammation presenting as immunoglobulin G4-related disease: a case report

**DOI:** 10.1186/1752-1947-5-427

**Published:** 2011-09-02

**Authors:** Kazuki Nagai, Kazuo Andoh, Noriko Nakamura, Katsumi Sakata

**Affiliations:** 1 Internal Medicine, Nagai Clinic, 1-7-25, Yokodai, Isogo-ku, Yokohama City, Kanagawa, 235-0045, Japan; 2 Department of Radiology, Saiseikai Yokohama-shi Nanbu Hospital, 3-2-10, Konandai, Konan-ku, Yokohama City, Kanagawa, 234-8503, Japan; 3 Department of Pathology, Saiseikai Yokohama-shi Nanbu Hospital, 3-2-10, Konandai, Konan-ku, Yokohama City, Kanagawa, 234-8503, Japan; 4 Department of Neurosurgery, Yokohama City University, 4-57, Urafune-cho, Minami-ku, Yokohama City, Kanagawa, 232-0024, Japan

## Abstract

**Introduction:**

Idiopathic sclerosing orbital inflammation is a rare and ill-defined heterogeneous entity, and a distinct subset of orbital inflammation. Recently, attention has been focused on immunoglobulin G4-related disease complicated with fibrotic changes in some other organs with high serum immunoglobulin G4 levels. This report presents a case of suspected idiopathic sclerosing orbital inflammation complicated with high serum immunoglobulin G4 levels.

**Case presentation:**

An 82-year-old Japanese woman had a 30-year history of chronic thyroiditis. She experienced right ptosis and eyelid swelling. These symptoms gradually developed over five years. The clinical and radiographic findings suggested that our patient had idiopathic sclerosing orbital inflammation. We were unable to obtain our patient's consent to perform a biopsy. While the serum immunoglobulin G level was within the normal limits, the serum immunoglobulin G4 level was significantly elevated. The serum immunoglobulin G4 levels decreased after the administration of oral prednisolone at a daily dose of 20 mg. In addition, the swelling and ptosis of the right upper eyelid disappeared gradually after four weeks. Our patient was then suspected to have idiopathic sclerosing orbital inflammation complicated with immunoglobulin G4-related disease and chronic thyroiditis.

**Conclusion:**

An orbital pseudotumor of this type is indicative of idiopathic sclerosing orbital inflammation immunoglobulin G4-related disease. Immunoglobulin G4 may thus be considered a subclass of immunoglobulin G when the serum immunoglobulin G level is within normal limits.

## Introduction

Idiopathic sclerosing orbital inflammation (ISOI) is a rare, distinct subset of orbital inflammation, which is difficult to diagnose and manage. In addition, ISOI is an ill-defined heterogeneous entity. There are many reports on ISOI [1-4]. Early intervention with immunosuppression in the form of corticosteroids can result in the control and even regression of the disease. Various diseases that cause fibrotic changes in different systemic organs have been reported in cases of ISOI [5,6]. Multifocal fibrosclerosis or systemic idiopathic fibrosis occurs when orbital pseudotumor (OPT) is complicated by idiopathic retroperitoneal fibrosis (IRF), sclerosing cholangitis (SC) or Riedel's thyroiditis [7,8]. This disease group has also been collectively known as "fibrotic overlap syndrome" due to its excellent response to steroids [9,10]. On the other hand, serum immunoglobulin (Ig) G4-elevated cases that are complicated with fibrotic changes in different systemic organs are known as IgG4-related autoimmune disease [11], hyper-IgG4 disease [12,13], and IgG4-related sclerosing disease [14-19]. Masaki *et al. *proposed a new clinical entity, IgG4-positive multiorgan lymph proliferative syndrome (MOLPS), based on the clinical features and a good response to steroids [20]. Thus far, however, the findings have been insufficient. In addition, no consensus has yet been reached regarding the definition of IgG4-related sclerosing disease. Recently, Umehara and Okazaki reached a consensus regarding its name and now call it "IgG4-related disease" [20].

An orbital pseudotumor as described in this case may indicate ISOI and may be relevant to IgG4-related disease. A subset of ISOI may include IgG4-related disease.

## Case presentation

An 82-year-old Japanese woman had a 30-year history of chronic thyroiditis. She had suffered from right ptosis and eyelid swelling for five years. She entered another hospital for a workup. However, she did not receive treatment at that time and her symptoms remained untreated. These symptoms were still persisting when she had a check-up at our clinic.

**Figure 1:**
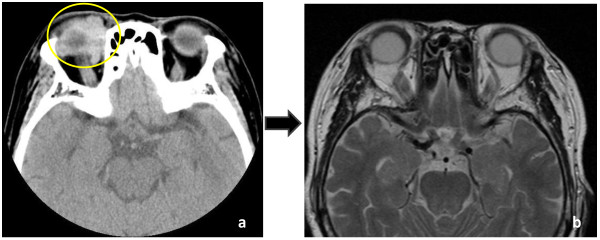
**Patient presented with swelling and ptosis of the right upper eyelid**. **(a) **Head CT of the orbit revealed abnormal high density area (yellow circle); **(b) **MRI revealed the presence of an infiltration of the right intraorbital diffuse mass which regressed and decreased in size one month after treatment was initiated.

Our patient had been administered levothyroxine sodium hydrate for several years. She had never received a blood transfusion, and her family history was unremarkable. A physical examination at the time of onset showed our patient to be well nourished; her height was 146 cm, and her weight was 52 kg. There was swelling and ptosis of her right upper eyelid. Her thyroid gland was not enlarged, but it was slightly hard when palpated. Her chest and heart were normal, and no abdominal tumor or hepatosplenomegaly were observed. There was also no remarkable swelling in her lymph nodes or peripheral edema in her extremities. Her neurologic function was also normal. No bilateral hilar lymphadenopathy (BHL) was identified in chest X-ray findings. Laboratory data were obtained during the check-up. Hematologic tests were within the normal limits. Her thyroid function tests showed free thyroxine 0.9 ng/dL (normal range 0.8-1.7 ng/dL), free tri-iodothyronine 2.2 pg/mL (normal range 2.2-4.1 pg/mL), thyroid stimulating hormone (TSH) 1.11 μIU/mL (normal range 0.35-3.73 μIU/mL), thyroid test × 6,400 (normal < × 100), anti-TSH-receptor antibody 9.5% (-15~15%), anti-thyroglobulin antibody 59.0 U/mL (normal range < 0.3 U/mL). Autoantibody tests were negative for rheumatoid arthritis, lupus erythematous, antinuclear antibody, antiDNA antibody and antimitochondrial antibody. Computerized tomography (CT) of her head was subsequently performed. A head CT of the orbit revealed an infiltration of the right intraorbital diffuse fibrotic tissues (Figure 1a).

**Figure 2:**
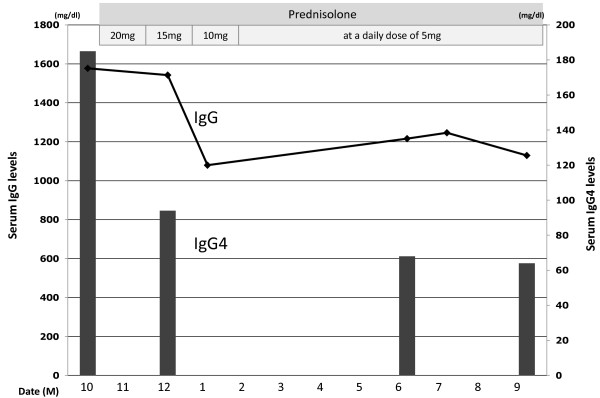
**Laboratory data before and after treatment with oral prednisolone**. Administration at a daily dose of 20 mg was initiated. The serum IgG4 levels decreased dramatically. The laboratory data initially showed an IgG4 level of 94 mg/dL. After nine months this had decreased to 64 mg/dL.

Our patient was followed for five years and showed continued swelling and ptosis of her right upper eyelid. Clinical and radiographic findings suggested her diagnosis to be ISOI complicated with chronic thyroiditis. While the serum IgG level was within the normal limits (1577; normal range 870-1700 mg/dL), the serum IgG4 level was found to be significantly elevated. The IgG subclass was measured resulting in a IgG1 level of 643 mg/dL (normal: 320-748 mg/dL), an IgG2 level of 701 mg/dL (normal: 208-754 mg/dL), an IgG3 level of 48.9 mg/dL (normal: 6.6-88.3 mg/dL), and an IgG4 level of 185 mg/dL (normal: 4.8-105 mg/dL). Therefore, our patient was diagnosed to have suspected ISOI complicated with high serum IgG4 levels and chronic thyroiditis. Oral prednisolone administration was initiated. The serum IgG4 levels decreased dramatically. The laboratory data showed IgG4 94 mg/dL (Figure 2). The swelling and ptosis of her right upper eyelid disappeared gradually. In addition, magnetic resonance imaging (MRI) revealed infiltration of the right intraorbital diffuse fibrotic tissues which regressed and diminished after four weeks (Figure 1b). After 12 months, the laboratory data showed IgG4 64 mg/dL. Our patient is currently healthy.

## Discussion

The OPT observed in this case was suspected to be ISOI and may have been related to IgG4-related disease. The diagnosis of IgG4-related disease is defined by both an elevated serum IgG4 level (> 135 mg/dL) and certain histopathological features, including lymphocyte and IgG4 positive plasma cell infiltration (ratio of IgG4 positive plasma cells to IgG positive plasma cells > 50% on a highly magnified slide checked at five points) [20]. In the present case, because a biopsy had not been performed, the diagnostic criteria for IgG4-related disease were not satisfied. However, despite the fact that the serum IgG level fell within the normal range, she had high levels of IgG4. Moreover, prednisolone was exceptionally effective, so an IgG4-related disease was suspected.

Neild reported that an increased IgG concentration in the absence of hypergammaglobulinemia is typical but, ideally, IgG4 should also be measured [14]. While the serum IgG level was within the normal limits in our patient, her serum IgG4 level was significantly elevated. It is prudent to measure blood IgG4 level in a case of ISOI with a normal IgG level. If the value is elevated, careful observation for the development of IRF and/or fibrotic changes in the other organs should be conducted. A strict follow-up is therefore recommended in such cases. Normally, when IgG4-related sclerosing disease is suspected, it is necessary to conduct a histopathological examination. However, in this case, we were unable to obtain the patient's consent to conduct a histopathological examination.

There are a few points to consider in this case. First of all, the question as to whether ISOI and chronic thyroiditis, which appeared as complications in the present case, were indeed related to IgG4 remains to be definitively concluded. To do this a histopathological study was required, but was not possible as we could not obtain the permission of our patient. However, ISOI itself is vaguely defined, and it was assumed to be an OPT accompanying fibrosis of unknown origin. One should take into account the possible chronic sclerosing dacryoadenitis in the case of ISOI [21]. Regarding the ISOI in the present case, chronic sclerosing dacryoadenitis, Graves' ophthalmopathy, etc., should also be included in the differential diagnosis. In Graves' ophthalmopathy, orbital CT and MRI findings show an enlargement of one or more extraocular muscles. However, the imaging of ISOI normally reveals an enlargement of one or more extraocular muscles with infiltration into the intraorbital fibrotic tissues. There are also no gold standard diagnostic criteria for differentiating ISOI from orbital lymphoid lesions or orbital cellulitis. Diagnosis in such cases is therefore based on the clinical presentation and response to treatment with some input from CT and MRI [22]. Taking the serology into consideration, based on the high values for IgG4, the eye symptoms in the present case were thus believed to indicate symptoms associated with IgG4-related disease.

Moreover, discriminating ISOI from sarcoidosis, Wegener's granulomatosis, lymphoma, cancer and other diseases is necessary. These possibilities should not be ruled out [23]. In the present case, differential antibodies were negative and BHL was not observed in her chest X-rays, so the abovementioned diseases could therefore be, to some extent, excluded. The lack of a pathological examination meant that they could not be definitively excluded.

The second important point to note is regards the thyroiditis. In the present case it was considered to be one type of chronic thyroiditis but, as with ISOI, the high values for IgG4 are suggestive of Riedel's thyroiditis. Riedel's thyroiditis is a chronic fibrosing disorder of unknown etiology, often associated with "multifocal fibrosclerosis." Riedel's thyroiditis is part of the IgG4-related systemic disease spectrum. In many cases, multifocal fibrosclerosis and IgG4-related systemic disease are probably the same entity [24]. In addition, this may be a variation of IgG4-related disease known as IgG4-related autoimmune disease, hyper-IgG4 disease, IgG4-related sclerosing disease, or IgG4-positive MOLPS. In the future, larger studies are called to elucidate the exact mechanism and clinical characteristics of this disorder.

## Conclusion

In cases of ISOI with high IgG level or a normal IgG level it is prudent to measure the blood IgG4 level. If the level rises, careful observation for the development of IRF, and/or fibrotic changes in the organs is necessary. A strict follow-up is recommended in such cases.

## Abbreviations

BHL: bilateral hilar lymphadenopathy; CT: computerized tomography; Ig: immunoglobulin; IRF: idiopathic retroperitoneal fibrosis; ISOI: idiopathic sclerosing orbital inflammation; MOLPS: multiorgan lymph proliferative syndrome; MRI: magnetic resonance imaging; OPT: orbital pseudotumor; SC: sclerosing cholangitis; TSH: thyroid stimulating hormone.

## Consent

Written informed consent was obtained from the patient for publication of this case report and any accompanying images. A copy of the written consent is available for review by the Editor-in-Chief of this journal.

## Competing interests

The authors declare that they have no competing interests.

## Authors' contributions

KN and KS drafted and wrote the manuscript and were involved in data interpretation. KN was involved in the care of our patient. KA provided CT images. NA was involved in administrative support. All authors read and approved the final manuscript.

## References

[B1] Chen YM Hu FR Liao SL Idiopathic sclerosing orbital inflammation--a case series study Ophthalmologica 2010 224 1 55 58 10.1159/000235564 19690442

[B2] Sahlin S Lignell B Williams M Dastmalchi M Orrego A Treatment of idiopathic sclerosing inflammation of the orbit (myositis) with infliximab Acta Ophthalmol 2009 87 8 906 908 10.1111/j.1755-3768.2008.01320.x 18631325

[B3] Brannan PA A review of sclerosing idiopathic orbital inflammation Curr Opin Ophthalmol 2007 18 5 402 404 10.1097/ICU.0b013e3282bfe85b 17700234

[B4] Zborowska B Ghabrial R Selva D McCluskey P Idiopathic orbital inflammation with extraorbital extension: case series and review Eye (Lond) 2006 20 1 107 113 10.1038/sj.eye.6701780 15920571

[B5] Fraile G Rodriguez-Garcia JL Moreno A Primary sclerosing cholangitis associated with systemic sclerosis Postgrad Med J 1991 67 784 189 192 10.1136/pgmj.67.784.189 2041852 PMC2398963

[B6] Khine AA Prabhakaran VC Selva D Idiopathic sclerosing orbital inflammation: two cases presenting with paresthesia Ophthal Plast Reconstr Surg 2009 25 1 65 67 10.1097/IOP.0b013e3181934624 19273935

[B7] Carton RW Wong R Multifocal fibrosclerosis manifested by vena caval obstruction and associated with vasculitis Ann Intern Med 1969 70 1 81 86 5763732 10.7326/0003-4819-70-1-81

[B8] Laitt RD Hubscher SG Darby S Elias E Sclerosing cholangitis associated with multifocal fibrosis: a case report GUT 1992 33 10 1430 1432 10.1136/gut.33.10.1430 1446876 PMC1379620

[B9] Mitchinson MJ The pathology of idiopathic retroperitoneal fibrosis J Clin Pathol 1970 23 8 681 689 10.1136/jcp.23.8.681 5488039 PMC476868

[B10] Comings DE Skubi KB Eyes JV Motulsky AG Familial multifocal fibrosclerosis. Findings suggesting that retroperitoneal fibrosis, mediastinal fibrosis, sclerosing cholangitis, Riedel's thyroiditis, and pseudotumor of the orbit may be different manifestations of a single disease Ann Intern Med 1967 66 5 884 892 6025229 10.7326/0003-4819-66-5-884

[B11] Kardar AH Sundin T AL-Suhaibani H Aslam M Peracha A Lindstedt E Successful treatment of idiopathic retroperitoneal fibrosis with steroids Ann Saudi Med 1997 17 4 419 422 17353593 10.5144/0256-4947.1997.419

[B12] Kamisawa T Funata N Hayashi Y Eishi Y Koike M Tsuruta K Okamoto A Egawa N Nakajima H A new clinicopathological entity of IgG4-related autoimmune disease J Gastroenterol 2003 38 10 982 984 10.1007/s00535-003-1175-y 14614606

[B13] Sugimoto T Morita Y Isshiki K Yamamoto T Uzu T Kashiwagi A Horie M Asai T Constrictive pericarditis as an emerging manifestation of hyper-IgG4 disease Int J Cardiol 2008 130 3 e100 101 10.1016/j.ijcard.2007.06.111 17727980

[B14] Neild GH Rodriguez-Justo M Wall C Connolly JO Hyper-IgG4 disease: report and characterisation of a new disease BMC Med 2006 4 23 10.1186/1741-7015-4-23 17026742 PMC1618394

[B15] Zen Y Harada K Sasaki M Sato Y Tsuneyama K Haratake J Kurumaya H Katayanagi K Masuda S Niwa H Morimoto H Miwa A Uchiyama A Portmann BC Nakanuma Y IgG4-related sclerosing cholangitis with and without hepatic inflammatory pseudotumor, and sclerosing pancreatitis-associated sclerosing cholangitis: do they belong to a spectrum of sclerosing pancreatitis? Am J Surg Pathol 2004 28 9 1193 1203 10.1097/01.pas.0000136449.37936.6c 15316319

[B16] Kamisawa T Nakajima H Egawa N Funata N Tsuruta K Okamoto A IgG4-related sclerosing disease incorporating sclerosing pancreatitis, cholangitis, sialadenitis and retroperitoneal fibrosis with lymphadenopathy Pancreatology 2006 6 1-2 132 137 10.1159/000090033 16327291

[B17] Kamisawa T Okamoto A Autoimmune pancreatitis: proposal of IgG4-related sclerosing disease J Gastroenterol 2006 41 7 613 625 10.1007/s00535-006-1862-6 16932997 PMC2780632

[B18] Nagai K Hosaka H Takahashi Y Kubo S Nakamura N Andoh K A case of IgG4-related sclerosing disease complicated with sclerosing cholangitis, idiopathic retroperitoneal fibrosis, and orbital pseudotumor BMJ Case reports 2009 10.1136/bcr.02.2009.1590 PMC3027777 21686984

[B19] Zen Y Inoue D Kitao A Onodera M Abo H Miyayama S Gabata T Matsui O Nakanuma Y IgG4-related Lung and Pleural Disease: A Clinicopathologic Study of 21 Cases Am J Surg Pathol 2009 33 12 1886 1893 10.1097/PAS.0b013e3181bd535b 19898222

[B20] Masaki Y Iwao H Nakajima A Miki M Sugai S Umehara H IgG4-related disease (IgG4+MOLPS) - diagnostic criteria and diagnostic problems Curr Immunol Rev 2011 7 172 177

[B21] Cheuk W Yuen HK Chan JK Chronic sclerosing dacryoadenitis: part of the spectrum of IgG4-related sclerosing disease? Am J Surg Pathol 2007 31 4 643 645 10.1097/01.pas.0000213445.08902.11 17414116

[B22] Kapur R Sepahdari AR Mafee MF Putterman AM Aakalu V Wendel LJA Setabutr P MR Imaging of orbital inflammatory syndrome, orbital cellulitis, and orbital lymphoid lesions: the role of diffusion-weighted imaging Am J Neuroradiol 2009 30 1 64 70 18842758 10.3174/ajnr.A1315 PMC3690287

[B23] Gordon LK A case of orbital inflammatory disease: a diagnostic and therapeutic challenge Eye 2006 20 1196 1206 10.1038/sj.eye.6702383 17019419

[B24] Dahlgren M Khosroshahi A Nielsen GP Dehpande V Stone JH Riedel's thyroiditis and multifocal fibrosclerosis are part of the IgG4-related systemic disease spectrum Arthritis Care Res 2010 62 9 1312 1318 10.1002/acr.20215 20506114

